# Focal concavity of posterior superior acetabulum and its relation with acetabular dysplasia and retroversion in adults without advanced hip osteoarthritis

**DOI:** 10.1186/s12891-015-0791-z

**Published:** 2015-11-02

**Authors:** Hirohito Tanaka, Keisuke Watarai, Iichiro Osawa, Michio Shiibashi, Yoon Taek Kim, Hiromi Oda, Hirohiko Azuma

**Affiliations:** Department of Orthopaedic Surgery, Saitama Medical University, 38 Morohongo, Moroyama-machi, Iruma-gun, Saitama 350–0495 Japan; Department of Radiology, Saitama Medical University, 38 Morohongo, Moroyama-machi, Iruma-gun, Saitama 350–0495 Japan; Information Technology Center, Saitama Medical University, 38 Morohongo, Moroyama-machi, Iruma-gun, Saitama 350–0495 Japan

**Keywords:** Acetabulum, Computed tomography, Focal concavity, Dysplasia, Retroversion

## Abstract

**Background:**

Although little is known, a limited number of three-dimensional computed tomography (CT) images of the pelvis present focal concavity of posterior superior acetabulum. The purpose of the present study was to investigate this morphologic deformity and its relation with dysplasia and retroversion in adults who were expected to have the original morphology of the acetabulum after growth.

**Methods:**

Consecutive adult patients with hip pain who visited our hospital and had three-dimensional pelvic CT images were retrospectively analyzed after approval of the institutional review board; exclusion criterions included diseases, injuries and operations that affect the morphology of the hip including radiographic osteoarthritis Tönnis grades 2 and 3. Focal concavity of posterior superior acetabulum was evaluated by three-dimensional CT image. Acetabular dysplasia was determined by lateral center edge (LCE) angle <25°, Tönnis angle >10°, and anterior center edge (ACE) angle <25° on standing hip radiographs. Acetabular version angle was measured at the one-fourth cranial level of axial CT image. A subgroup analysis included only younger adult patients up to 50 years.

**Results:**

The subjects analyzed were 46 men (92 hips) and 54 women (108 hips) with a median age of 57.5 (21–79) and 51.0 (26–77) years, respectively. Focal concavity of posterior superior acetabulum was observed in 13 hips; 7 patients had unilaterally, while 3 patients showed bilaterally. Among these hips, pain was observed in 8 hips but 4 hips (2 patients) were associated with injuries. This morphologic abnormality was not associated with acetabular dysplasia determined by LCE angle <25°, Tönnis angle >10° or ACE angle <25°. Of note, no acetabulum with the deformity plus dysplasia was retroverted. These findings were confirmed in a subgroup analysis including 22 men (44 hips) and 27 women (54 hips) with a median age of 31.0 (21–50) and 41.0 (26–50) years, respectively.

**Conclusions:**

Focal concavity of posterior superior acetabulum could be a rare morphologic abnormality of acetabular formation independent of lateral or anterior dysplasia or retroversion.

**Electronic supplementary material:**

The online version of this article (doi:10.1186/s12891-015-0791-z) contains supplementary material, which is available to authorized users.

## Background

In 1999, Reynolds et al. described retroversion of the acetabulum as a solitary anomaly that could result in hip pain [1]. It is now generally accepted that acetabular retroversion is a cause of painful femoro-acetabular impingement [2, 3].

It has been consistently reported that patients with acetabular dysplasia have higher frequency of acetabular retroversion if a cross-over sign on the anteroposterior radiograph of the pelvis is used for the diagnosis [4, 5], while recent data have also suggested the differences between dysplasia and retroversion of the acetabulum. For example, Tannast et al. [6] showed that pelvic morphology differed in rotation and obliquity between acetabular retroversion and developmental dysplasia, and Tannenbaum et al. [3] found that the frequency of acetabular retroversion was higher in men compared to women in contrast to acetabular dysplasia.

**Fig. 1:**
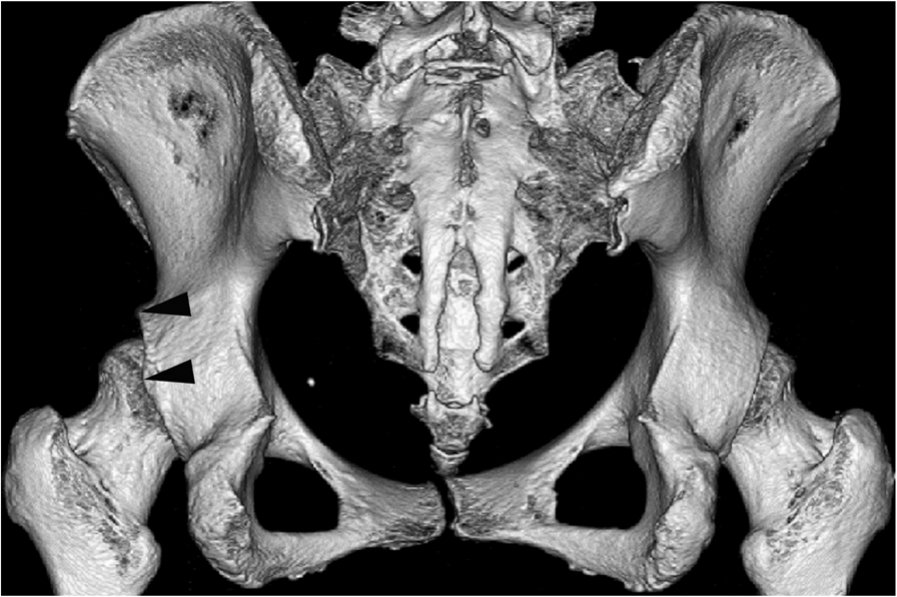
A posterior view of three-dimensional pelvic CT image in a 52-year-old woman, showing focal concavity of posterior superior acetabulum as indicated by two arrows

There are few reports assessing the original morphology of the adult acetabulum with dysplasia without advanced hip osteoarthritis [7]. We have observed that a small number of three-dimensional computed tomography (CT) images of the pelvis present focal concavity of posterior superior acetabulum (Fig. 1 and Additional file 1: Figure S1; unpublished data). To our knowledge, however, this morphologic abnormality has not yet been studied. The present study retrospectively investigated the focal deformity and its relation with dysplasia and retroversion in adults without diseases, injuries or operations that affect the morphology of the hip.

## Methods

### Subject selection

In the present study, we included adults less than 80 years old from consecutive patients with hip pain who visited our hospital and had three-dimensional CT images of the pelvis from January 2010 to August 2012. We excluded patients without standing pelvic radiographs of the anteroposterior and false profile views or with radiographic hip osteoarthritis Tönnis grades 2 and 3; mild hip osteoarthritis (Tönnis grade 1) was judged to be acceptable for the analysis of original morphology. Patients were also excluded if they had a history of hip fracture or surgery and diseases that affect the morphology of the hip including osteonecrosis of the femoral head and rheumatoid arthritis, or if CT images limited to measure angles precisely because of poor positioning and there were no raw data available to recreate reconstructed images. In addition to the analysis of all subjects, we performed a subgroup analysis that was limited to only younger adult patients up to 50 years to further focus on the original morphology after growth. The institutional review board of the Saitama Medical University Hospital approved the present study (approval No. 13-047-1); informed consent was waived because of the retrospective design.

### Plain radiograph acquisition

Standing anteroposterior radiographs of the hip were made with the limbs parallel and with the feet internally rotated approximately 20°. The central beam was directed to the midpoint between the superior border of the pubic symphysis and the center of a line connecting both anterior superior iliac spines, at a distance of 120 cm from the film. False-profile radiographs of the hip were obtained in a standing position. Affected hip was positioned against the film cassette, with the ipsilateral foot parallel to the cassette stand. The pelvis was rotated 65° relative to the cassette. The x-ray beam was directed toward the center of the femoral head at a tube-to-film distance of 120 cm.

### CT image acquisition

All CT images were acquired with a 16-slice or 128-slice multidetector CT scanner system (Somatom Emotion 16 or Somatom Difinition Flash; Siemens Healthcare, Forchheim, Germany). The scan parameters for the 16-slice CT scanner were tube voltage 130 kV, reference mAs 140 mAs, collimation 1×16×0.6 mm, gantry rotation time 0.6 s, pitch 0.9, pixel matrix size 512×512, and those for the 128-slice CT were tube voltage 120 kV, reference mAs 185 mAs, collimation 2×64×0.6 mm, gantry rotation time 1.0 s, pitch 0.8, pixel matrix size 512×512. Automatic exposure control (CARE Dose 4D, Siemens Healthcare, Forchheim, Germany) was activated in all scans. For a given reference mAs, this technique can adjust the tube current in real-time to optimize radiation dose utilization. The radiation doses of all patients were recorded; the average CT dose index volume (CTDI_vol_) on 16-slice and 128-slice CT was approximately 12 mGy and 8 mGy, respectively, while the corresponding dose-length product (DLP) was approximately 375 mGy*cm and 238 mGy*cm. Patients were placed spine with the limbs parallel and with enough internal rotation for the feet to touch each other. Images were obtained from anterior superior iliac spines to the proximal portion of the femurs. Axial and coronal images were reconstructed at 3-mm slice thickness using filtered back projection. Three-dimensional volume-rendered images were acquired with a 0.75-mm reconstructed slice thickness and a 0.5-mm reconstruction increment, on Aquarius iNtuition 3D workstation (TeraRecon, Foster City, CA, USA).

### Image analysis

Focal concavity of posterior superior acetabulum (Fig. 1 and Additional file 1: Figure S1) was evaluated by three-dimensional CT image of the pelvis and the selection was performed under the agreement of all authors. Acetabular dysplasia was determined by not only lateral center edge (LCE) angle <25° on standing anteroposterior radiographs, but also Tönnis angle >10° and anterior center edge (ACE) angle <25° on standing radiographs of the anteroposterior and false-profile views [8], respectively. LCE angle was formed by a vertical line through the center of the femoral head and a second line through the lateral edge of the acetabulum to the center of the femoral head. Tönnis angle was created by a horizontal line and a line connecting the lateral and inferior aspects of the acetabular sourcil. ACE angle was composed of a vertical line through the center of the femoral head and a second line through the most anterior point of the acetabulum to the center of the femoral head. Acetabular retroversion was judged by version angle <0° at the one-fourth cranial level of the acetabulum in an axial CT image according to a recent validation study [9]; we did not use cross-over sign because recent studies suggest that it might not provide the accurate diagnosis of acetabular retroversion [10, 11]. This angle was formed by a reference line which is perpendicular to a horizontal line connecting the posterior margins of both acetabuli, and a line connecting the anterior and posterior margins of the acetabulum. Two authors (HT and KW) with more than 10 years of experience in this field performed all measurements independently and their mean values were used for the analyses after confirming the inter-rater reliability shown in Additional files 2: Table S1, 3: Figures S2 and 4: Figure S3.

### Statistical analysis

Comparisons of continuous variables for two groups and associations between categorical variables were analyzed by Mann–Whitney *U* test and Fisher’s exact test, respectively, using StatMate v4.01 (ATMS Co., Ltd., Tokyo, Japan). A *p*-value of <0.05 was considered statistically significant.

## Results

### All subjects

Among 488 patients selected according to the inclusion criterions, we excluded those without standing pelvic radiograph of the false profile view (*n* = 283) and with radiographic hip osteoarthritis Tönnis grades 2 and 3 (*n* = 152), a history of hip fracture (*n* = 121) or surgery (*n* = 125), diseases that affect the morphology of the hip (*n* = 75) and inappropriate CT images (*n* = 26). The numbers of patients excluded in these criterions overlap and subjects analyzed in the present study were a total of 100 patients (200 hips). There were 46 men (92 hips) and 54 women (108 hips) with a median age of 57.5 (21 to 79) and 51.0 (26 to 77) years, respectively.

Focal concavity of posterior superior acetabulum was observed in a total of 13 hips (6.5 %); 7 patients had unilaterally (3 hips with pain and 4 hips without pain) while 3 patients showed bilaterally (5 hips with pain and 1 hip without pain), as shown in Additional files 5: Figures S4 and 6: S5. Among the 8 hips with pain, however, 4 hips (2 patients) were associated with injuries. Acetabular dysplasia determined by LCE angle <25°, Tönnis angle >10° and ACE angle <25° included 45.5, 44.5 and 34.0 %, respectively, while 12.0 % had acetabular retroversion (Table 1).

**Table 1:** Characteristics of all subjects

	All	Male	Female	*p*-value*
Patient (*n*)	100	46	54	
Acetabulum (*n*)	200	92	108	
Age				
Mean (SD) (year)^a^	51.0 (16.0)	50.6 (18.6)	51.4 (13.5)	0.884
Median (year)	52.0	57.5	51.0	
Range (year)	21–79	21–79	26‐77	
Focal concavity of posterior superior acetabulum (*n*)	13/200	7/92	6/108	0.578
Lateral center edge angle				
Mean (SD) (°)^a^	25.1 (8.8)	27.9 (6.7)	22.7 (9.7)	<0.001
Range (°)	2.4–45.0	10.5–45.0	2.4–44.3	
Dysplasia (<25°) (*n*)^b^	91/200	28/92	63/108	<0.001
Tönnis angle				
Mean (SD) (°)^a^	9.5 (6.9)	7.2 (5.7)	11.5 (7.2)	<0.001
Range (°)	−6.7–29.6	−6.7–23.2	−6.0–29.6	
Dysplasia (>10°) (*n*)^b^	89/200	28/92	61/108	<0.001
Anterior center edge angle				
Mean (SD) (°)^a^	27.9 (10.3)	31.3 (7.9)	24.9 (11.1)	<0.001
Range (°)	−10.3–50.5	11.6–49.6	−10.3–50.5	
Dysplasia (<25°) (*n*)^b^	68/200	19/92	49/108	<0.001
Acetabular version angle				
Mean (SD) (°)^a^	12.9 (9.7)	8.6 (10.0)	16.5 (7.8)	<0.001
Range (°)	−10.5–38.5	−10.5–38.5	−1.0–36.5	
Retroversion (<0°) (*n*)^b^	24/200	22/92	2/108	<0.001

*Comparison between male and female values

^a^Mann-Whitney *U* test

^b^Fisher’s exact test

There was no gender- or age-related difference in focal concavity of posterior superior acetabulum. In contrast, the frequency of acetabular dysplasia was higher in women while that of acetabular retroversion was higher in men (Table 1); notably, men had 22 retroverted acetabuli (23.9 %) but women had only 2 retroverted acetabuli (1.9 %). Patients with retroverted acetabuli were younger than those with anteverted acetabuli (Table 2).

**Table 2:** Relation between patient age and focal concavity of posterior superior acetabulum, acetabular dysplasia or acetabular retroversion in all subjects

	Positive	Negative	*p*-value*
Focal concavity of posterior superior acetabulum			
Mean (SD) (year)	49.8 (16.8)	51.1 (16.0)	0.705
Range (year)	28–79	21–77	
Lateral center edge angle <25°			
Mean (SD) (year)	48.8 (13.8)	52.9 (17.5)	0.045
Range (year)	22–77	21–79	
Tönnis angle >10°			
Mean (SD) (year)	51.8 (14.0)	50.4 (17.5)	0.689
Range (year)	23–77	21–79	
Anterior center edge angle <25°			
Mean (SD) (year)	47.9 (14.1)	52.6 (16.7)	0.034
Range (year)	21–77	21–79	
Acetabular version angle <0°			
Mean (SD) (year)	42.5 (19.1)	52.2 (15.2)	0.011
Range (year)	22–74	21–79	

^*^Mann–Whitney *U* test

**Fig. 2:**
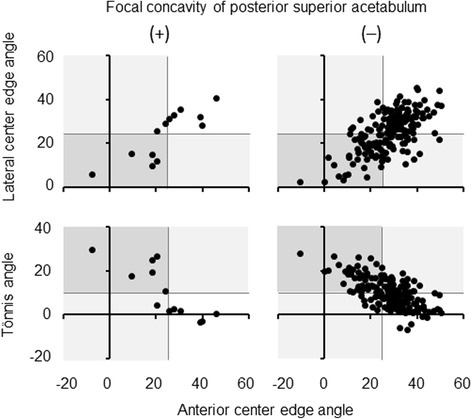
Relation between focal concavity of posterior superior acetabulum and acetabular dysplasia in all subjects. Focal concavity of posterior superior acetabulum was evaluated by three-dimensional CT image. Acetabular dysplasia was determined by lateral center edge angle <25°, Tönnis angle >10°, or anterior center edge angle <25° on standing pelvic radiographs

**Fig. 3:**
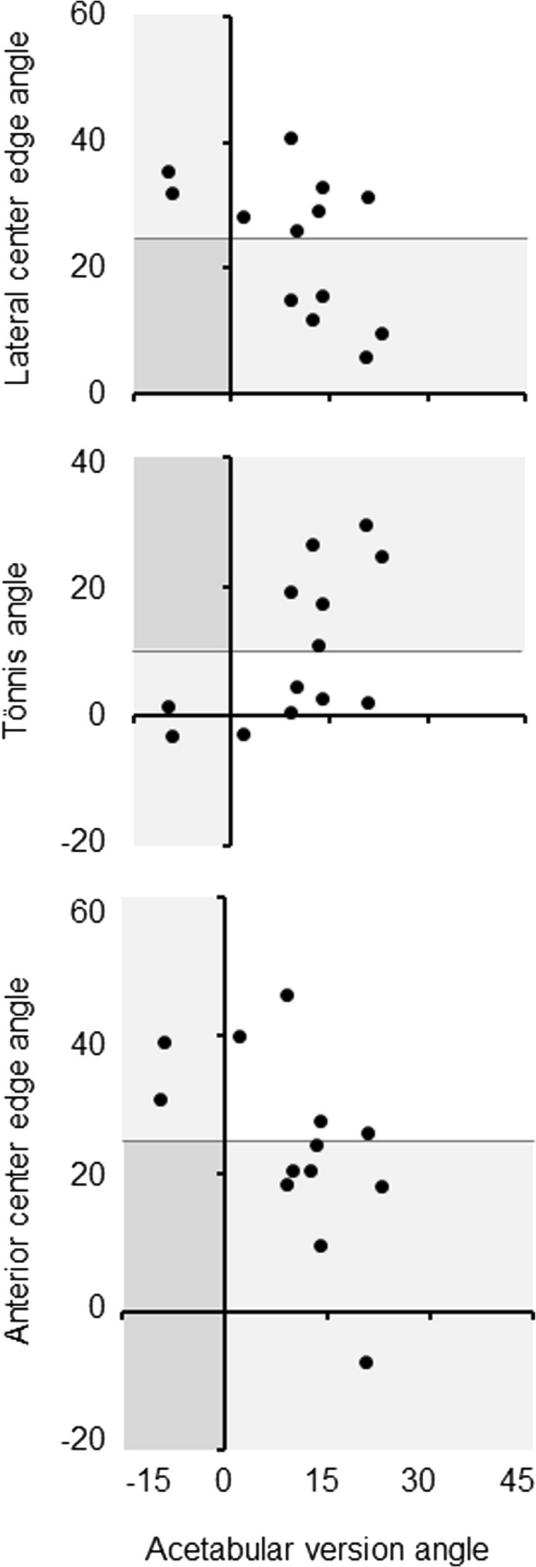
Relation between acetabular dysplasia and retroversion in all subjects with focal concavity of posterior superior acetabulum. Focal concavity of posterior superior acetabulum was evaluated by three-dimensional CT image. Acetabular dysplasia was determined by lateral center edge angle <25°, Tönnis angle >10°, or anterior center edge angle <25° on standing pelvic radiographs. Acetabular retroversion was judged by version angle <0° at the one-fourth cranial level of axial CT image

Focal concavity of posterior superior acetabulum was not associated with acetabular dysplasia determined by LCE angle <25°, Tönnis angle >10° or ACE angle <25° (Table 3, Fig. 2). Of note, no acetabulum with this morphologic abnormality plus dysplasia was retroverted (Table 4, Fig. 3).

**Table 3:** Relation between focal concavity of posterior superior acetabulum and acetabular dysplasia in all subjects

	Focal concavity of posterior superior acetabulum		
	(+)	(−)	*p*-value*
Acetabular dysplasia (*n*, %)			
Lateral center edge angle <25°	5/13, 38.5	86/187, 46.0	0.775
Tönnis angle >10°	6/13, 46.2	83/187, 44.4	1.000
Anterior center edge angle <25°	7/13, 53.8	61/187, 32.6	0.136

*Fisher’s exact test

**Table 4:** Relation between acetabular dysplasia and retroversion in all subjects with focal concavity of posterior superior acetabulum

	Retroversion	Anteversion	*p*-value*
Acetabular dysplasia (*n*, %)			
Lateral center edge angle <25°	0/13, 0.0	5/13, 38.5	0.020
Tönnis angle >10°	0/13, 0.0	6/13, 46.2	0.015
Anterior center edge angle <25°	0/13, 0.0	7/13, 53.8	0.003

*Fisher’s exact test

### Subjects at 50 years or younger

There were 22 men (44 hips) and 27 women (54 hips) with a median age of 31.0 (21 to 50) and 41.0 (26 to 50) years, respectively. A total of 7 hips (7.1 %) had focal concavity of posterior superior acetabulum; 3 patients had unilaterally (3 hips without pain) while 2 patients showed bilaterally (3 hips with pain and 1 hip without pain), as shown in Additional file 5: Figure S4. Among the 3 hips with pain, 2 hips (1 patient) were associated with an injury. Acetabular dysplasia determined by LCE angle <25°, Tönnis angle >10° and ACE angle <25° included 53.1, 43.9 and 38.8 %, respectively, while 16.3 % had acetabular retroversion (Table 5).

**Table 5:** Characteristics of subjects at 50 years or younger

	All	Male	Female	*p*-value*
Patient (*n*)	49	22	27	
Acetabulum (*n*)	98	44	54	
Age				
Mean (SD) (year)^a^	36.8 (8.7)	32.9 (9.0)	40.0 (6.8)	<0.001
Median (year)	38.0	31.0	41.0	
Range (year)	21–50	21–50	26–50	
Focal concavity of posterior				
superior acetabulum (*n*)	7/98	3/44	4/54	1.000
Lateral center edge angle				
Mean (SD) (°)^a^	23.6 (9.3)	27.6 (6.9)	20.3 (9.7)	<0.001
Range (°)	2.4–45.0	14.0–45.0	2.4–41.5	
Dysplasia (<25°) (*n*)^b^	52/98	15/44	37/54	0.001
Tönnis angle				
Mean (SD) (°)^a^	9.5 (7.6)	6.8 (6.4)	11.6 (7.8)	0.003
Range (°)	−6.7–27.5	−6.7–23.2	−6.0–27.5	
Dysplasia (>10°) (*n*)^b^	43/98	11/44	32/54	<0.001
Anterior center edge angle				
Mean (SD) (°)^a^	27.8 (11.6)	31.9 (8.8)	24.5 (12.4)	0.002
Range (°)	−10.3–50.5	11.6–49.6	−10.3–50.5	
Dysplasia (<25°) (*n*)^b^	38/98	10/44	28/54	0.004
Acetabular version angle				
Mean (SD) (°)^a^	11.5 (9.8)	6.6 (10.4)	15.4 (7.0)	<0.001
Range (°)	−10.0–38.5	−10.0–38.5	−1.0–29.0	
Retroversion (<0°) (*n*)^b^	16/98	15/44	1/54	<0.001

*Comparison between male and female values

^a^Mann-Whitney *U* test

^b^Fisher’s exact test

No gender- or age-related difference in focal concavity of posterior superior acetabulum was observed. In contrast, the frequency of acetabular dysplasia was higher in women and that of acetabular retroversion was higher in men (Table 5); men had 15 retroverted acetabuli (34.1 %) while women had only 1 retroverted acetabuli (1.9 %). Patients with retroverted acetabuli were younger than those with anteverted acetabuli (Table 6).

**Table 6:** Relation between patient age and focal concavity of posterior superior acetabulum, acetabular dysplasia or acetabular retroversion in subjects at 50 years or younger

	Positive	Negative	*p*-value*
Focal concavity of posterior superior acetabulum			
Mean (SD) (year)	37.4 (9.2)	36.7 (8.6)	0.841
Range (year)	28–50	21–50	
Lateral center edge angle <25°			
Mean (SD) (year)	38.6 (7.3)	34.7 (9.5)	0.030
Range (year)	22–50	21–50	
Tönnis angle >10°			
Mean (SD) (year)	39.4 (7.3)	34.8 (9.1)	0.010
Range (year)	23–50	21–50	
Anterior center edge angle <25°			
Mean (SD) (year)	37.5 (8.0)	36.3 (9.0)	0.500
Range (year)	21–50	21–50	
Acetabular version angle <0°			
Mean (SD) (year)	29.5 (5.3)	38.2 (8.5)	0.009
Range (year)	22–39	21–50	

^*^Mann–Whitney *U* test

**Fig. 4:**
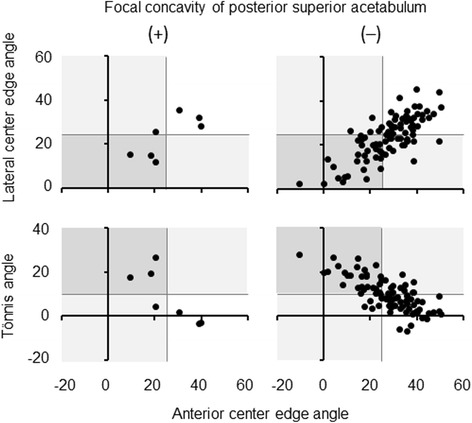
Relation between focal concavity of posterior superior acetabulum and acetabular dysplasia in subjects at 50 years or younger. Focal concavity of posterior superior acetabulum was evaluated by three-dimensional CT image. Acetabular dysplasia was determined by lateral center edge angle <25°, Tönnis angle >10°, or anterior center edge angle <25° on standing pelvic radiographs

**Fig. 5:**
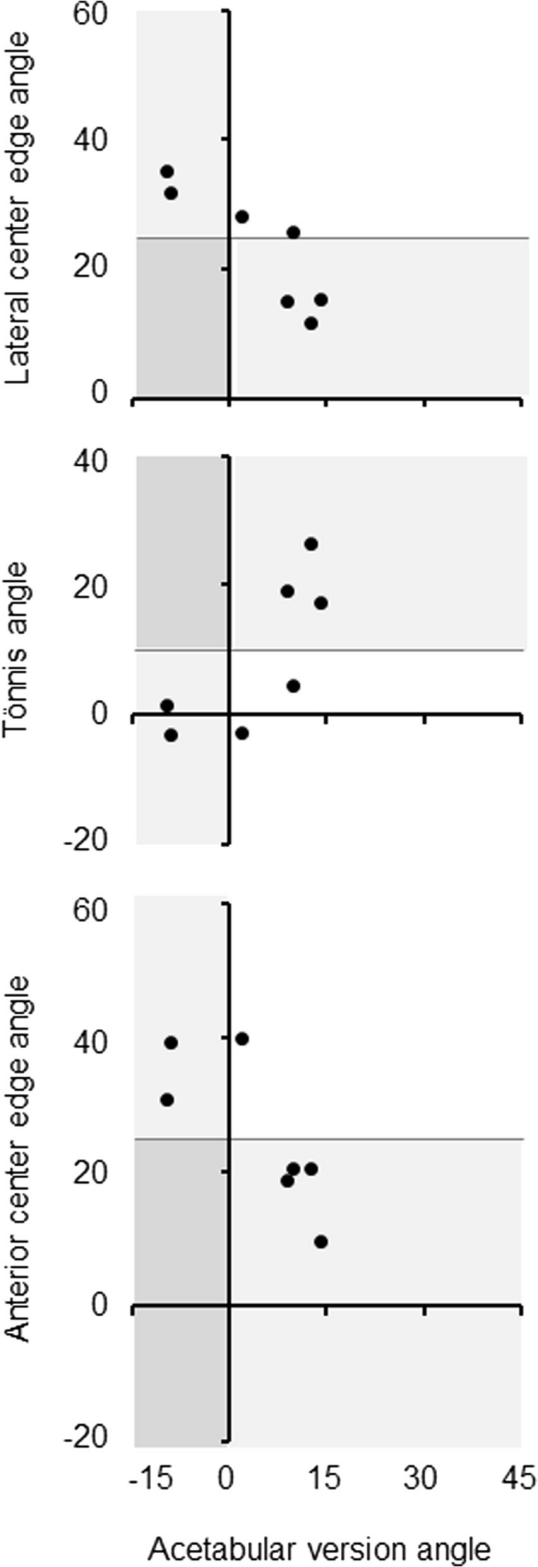
Relation between acetabular dysplasia and retroversion in subjects at 50 years or younger with focal concavity of posterior superior acetabulum. Focal concavity of posterior superior acetabulum was evaluated by three-dimensional CT image. Acetabular dysplasia was determined by lateral center edge angle <25°, Tönnis angle >10°, or anterior center edge angle <25° on standing pelvic radiographs. Acetabular retroversion was judged by version angle <0° at the one-fourth cranial level of axial CT image

Focal concavity of posterior superior acetabulum was not linked to acetabular dysplasia determined by LCE angle <25°, Tönnis angle >10° or ACE angle <25° (Table 7, Fig. 4). No acetabulum with this focal deformity plus dysplasia was retroverted (Table 8, Fig. 5).

**Table 7:** Relation between focal concavity of posterior superior acetabulum and acetabular dysplasia in subjects at 50 years or younger

	Focal concavity of posterior superior acetabulum		
	(+)	(−)	*p*-value*
Acetabular dysplasia (*n*, %)			
Lateral center edge angle <25°	3/7, 42.9	49/91, 53.8	0.703
Tönnis angle >10°	3/7, 42.9	40/91, 44.0	1.000
Anterior center edge angle <25°	4/7, 57.1	34/91, 37.4	0.425

*Fisher’s exact test

**Table 8:** Relation between acetabular dysplasia and retroversion in subjects at 50 years or younger with focal concavity of posterior superior acetabulum

	Retroversion	Anteversion	*p*-value*
Acetabular dysplasia (*n*, %)			
Lateral center edge angle <25°	0/7, 0.0	3/7, 42.9	0.192
Tönnis angle >10°	0/7, 0.0	3/7, 42.9	0.192
Anterior center edge angle <25°	0/7, 0.0	4/7, 57.1	0.003

*Fisher’s exact test

## Discussion

The present study investigated adult patients without diseases, injuries or operations that affect the morphology of the hip including radiographic osteoarthritis Tönnis grades 2 and 3. As a result, focal concavity of posterior superior acetabulum was observed in 6.5 % of 200 hips in 46 men (92 hips) and 54 women (108 hips) with a median age of 57.5 (21 to 79) and 51.0 (26 to 77) years, respectively. A similar frequency (7.1 % in 98 hips) of this deformity was confirmed by a subgroup analysis including 22 men (44 hips) and 27 women (54 hips) with a median age of 31.0 (21 to 50) and 41.0 (26 to 50) years, respectively. All subjects had hip pain unilaterally or bilaterally and it was unclear whether the morphologic abnormality can result in hip pain. This focal deformity did not show any specific feature regarding gender or age, while there are marked gender- and age-related differences in dysplasia and retroversion of the acetabulum.

Focal concavity of posterior superior acetabulum was not associated with lateral or anterior acetabular dysplasia determined by LCE angle <25°, Tönnis angle >10° or ACE angle <25°, or acetabular retroversion measured at the one-fourth cranial level of axial CT image. These results might be compatible with previous reports suggesting that the original morphology of acetabular dysplasia has a wide variety of deficiency types [7] and that there are differences between dysplasia and retroversion of the acetabulum [3, 6].

In agreement with the finding by Tannenbaum et al. [3], the present data showed that men had more retroverted acetabuli; although little is known, this apparent gender-related difference might be linked to the observation that external rotation of the lower limbs was more common in boys before birth [12]. The data presented also confirm that acetabular retroversion was associated with earlier onset of hip pain, as previously reported [5]. The consistency between our results and others [3, 5] could support that the present subjects were properly selected. From a diagnostic point of view, acetabular retroversion can be one cause of hip pain, potentially relating to femoro-acetabular impingement, especially in younger men, while such possibility might be low when focal concavity of posterior superior acetabulum as well as lateral or anterior acetabular dysplasia exists because no acetabulum with this morphologic abnormality plus dysplasia was retroverted.

The acetabulum is formed by ilium, ischium and pubis during growth and focal concavity of posterior superior acetabulum could be one hypoplastic deformity of acetabular wall. Indeed, it appears that the region of this deformity corresponds to the ilium (Additional file 7: Figure S6), possibly resulting from the relative growth disturbance compared to the ischium developmentally. If correct, acetabular retroversion [1, 12, 13] might be associated with congenital mal-orientation, because all acetabuli with the morphologic abnormality plus dysplasia were not retroverted. The hypothesis would be consistent with the facts that the position of a fetus in an uterus can influence acetabular morphology [12] and acetabular version angle at the one-fourth cranial level increases with growth [14].

The present study has several limitations. There is certain selection bias due to the way patients were selected for this retrospective review; non-patient volunteers or patients without hip pain were not available due to practical difficulties including the radiation dose of three-dimensional CT. Accordingly, the present results cannot be applied to general population. Another methodological issue could be consensus interpretation in imaging research [15]. Analyzing all three-dimensional CT images, acquired by two types of CT scanners, together might also cause difficulties with interpretation.

## Conclusions

In adult patients who were expected to have the original morphology of the acetabulum after growth, focal concavity of posterior superior acetabulum was observed in 13 hips (6.5 % of 200 hips). Among these hips, pain was observed in 8 hips (61.5 %), though 4 hips (2 patients) were associated with injuries. This focal deformity could be a morphologic abnormality of acetabular formation that is independent of lateral or anterior dysplasia or retroversion.

## Additional files

Additional file 1: Figure S1. Axial, coronal and sagittal two-dimensional pelvic CT images in a 52-year-old woman, showing focal concavity of posterior superior acetabulum as indicated by arrows. These images were from the same patient as the image in Figure 1. (TIFF 463 kb) Additional file 2: Table S1. Inter-rater reliability between two readers. (DOCX 21 kb) Additional file 3: Figure S2. Inter-rater reliability between two readers of lateral center edge angle, Tönnis angle, anterior center edge angle, and acetabular version angle in all subjects. (TIFF 42 kb) Additional file 4: Figure S3. Inter-rater reliability between two readers of lateral center edge angle, Tönnis angle, anterior center edge angle, and acetabular version angle in subjects at 50 years or younger. (TIFF 42 kb) Additional file 5: Figure S4. A posterior view of three-dimensional pelvic CT image in all subjects at 50 years or younger with focal concavity of posterior superior acetabulum as indicated by arrows. (TIFF 406 kb) Additional file 6: Figure S5. A posterior view of three-dimensional pelvic CT image in all subjects at 51 years or older with focal concavity of posterior superior acetabulum as indicated by arrows. (TIFF 443 kb) Additional file 7: Figure S6. A posterior view of the representative three-dimensional pelvic CT image during growth, showing the fusion site between ilium and ischium. (TIFF 157 kb)

## Abbreviations

CT: Computed tomography
LCE: Lateral center edge
ACE: Anterior center edge
