# Supplementary figures and images for: Focal concavity of posterior superior acetabulum and its relation with acetabular dysplasia and retroversion in adults without advanced hip osteoarthritis
Source: BMC Musculoskelet Disord. 2015 Nov 2;16:330. doi: 10.1186/s12891-015-0791-z (PMC4631111; doi:10.1186/s12891-015-0791-z)

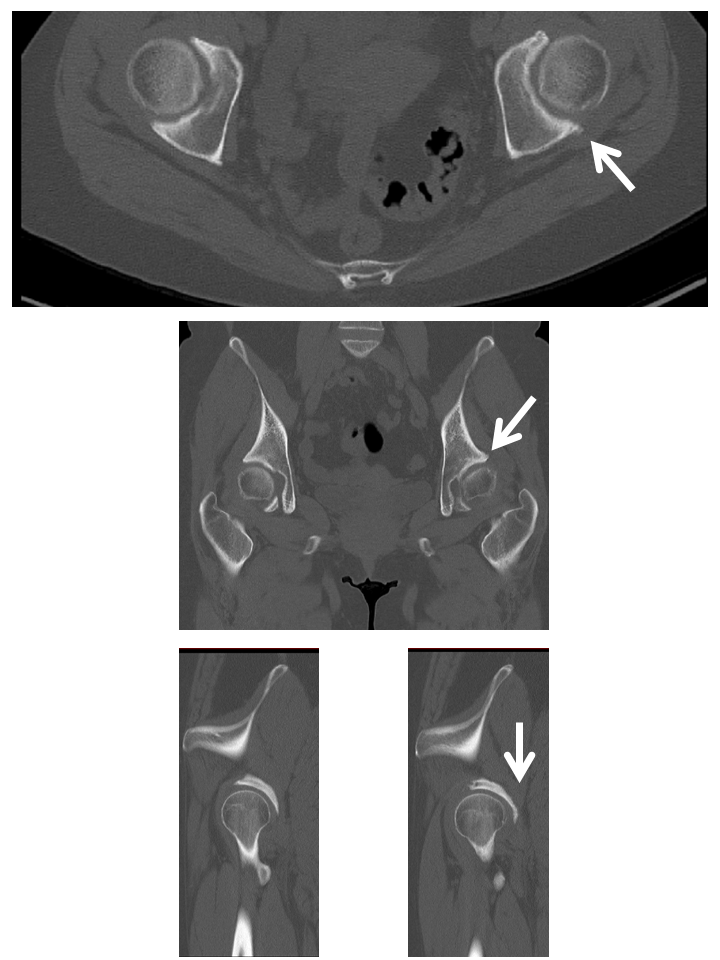

Supplement: Additional file 1: Figure S1. — Axial, coronal and sagittal two-dimensional pelvic CT images in a 52-year-old woman, showing focal concavity of posterior superior acetabulum as indicated by arrows. These images were from the same patient as the image in Figure 1. (TIFF 463 kb) [file 12891_2015_791_MOESM1_ESM.tif]

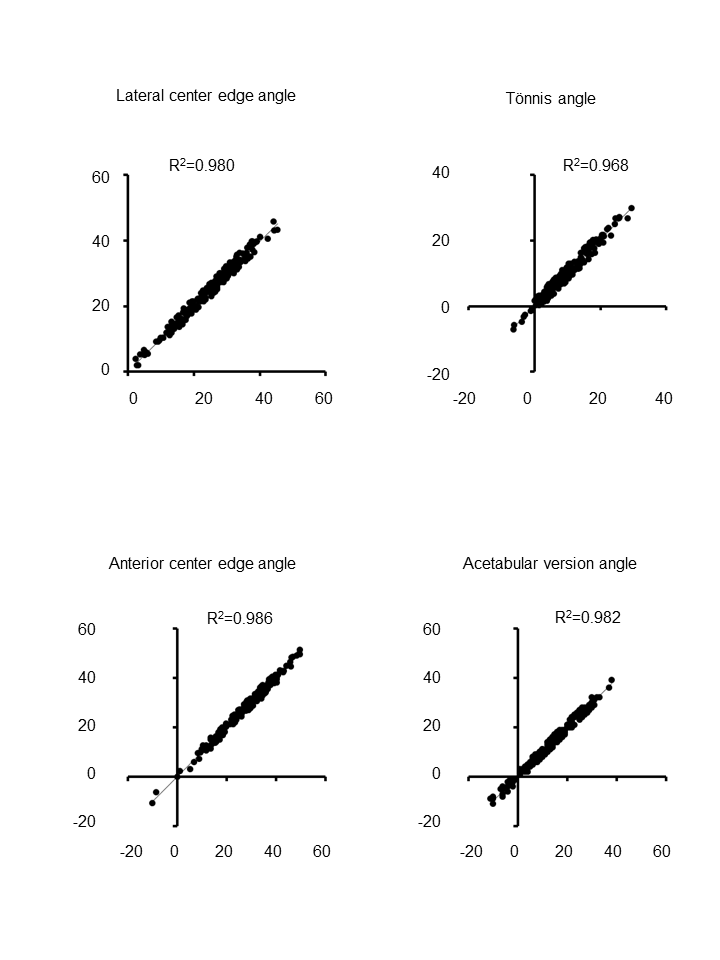

Supplement: Additional file 3: Figure S2. — Inter-rater reliability between two readers of lateral center edge angle, Tönnis angle, anterior center edge angle, and acetabular version angle in all subjects. (TIFF 42 kb) [file 12891_2015_791_MOESM3_ESM.tif]

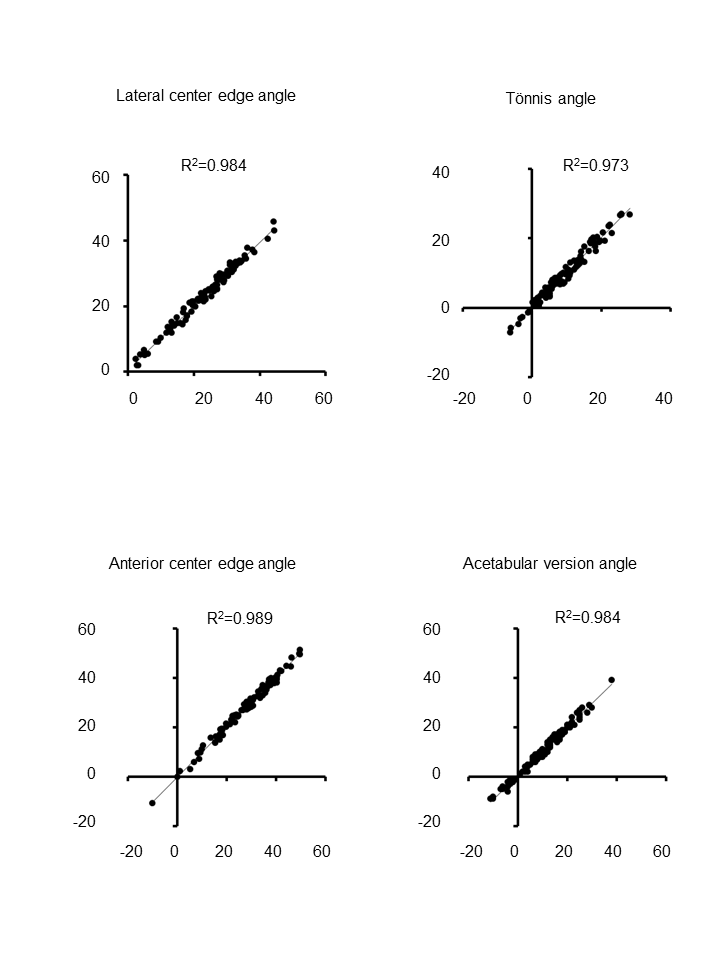

Supplement: Additional file 4: Figure S3. — Inter-rater reliability between two readers of lateral center edge angle, Tönnis angle, anterior center edge angle, and acetabular version angle in subjects at 50 years or younger. (TIFF 42 kb) [file 12891_2015_791_MOESM4_ESM.tif]

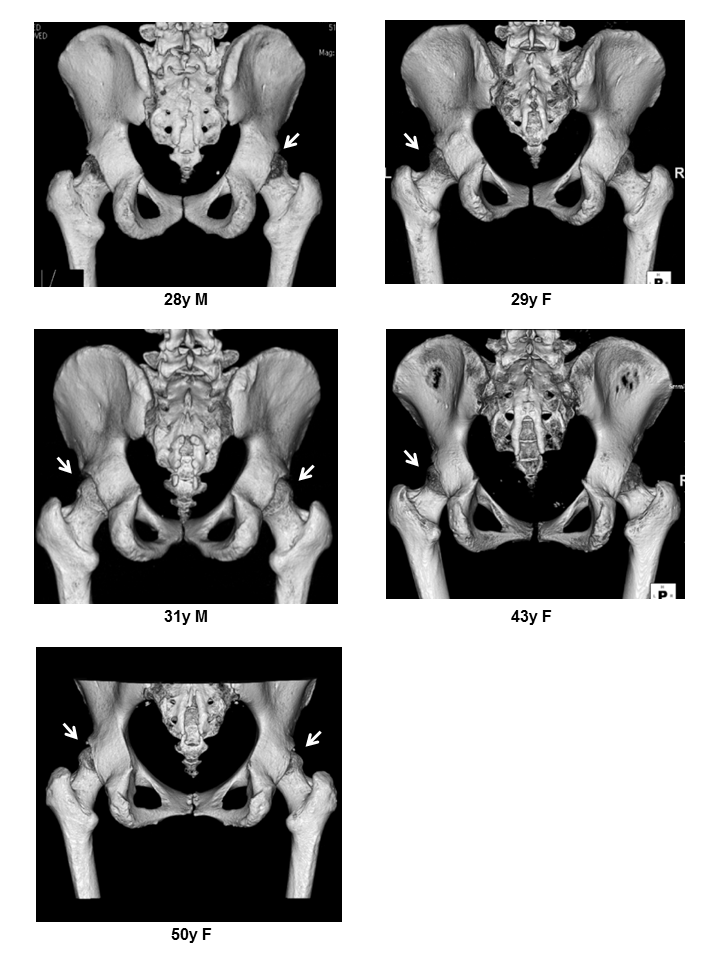

Supplement: Additional file 5: Figure S4. — A posterior view of three-dimensional pelvic CT image in all subjects at 50 years or younger with focal concavity of posterior superior acetabulum as indicated by arrows. (TIFF 406 kb) [file 12891_2015_791_MOESM5_ESM.tif]

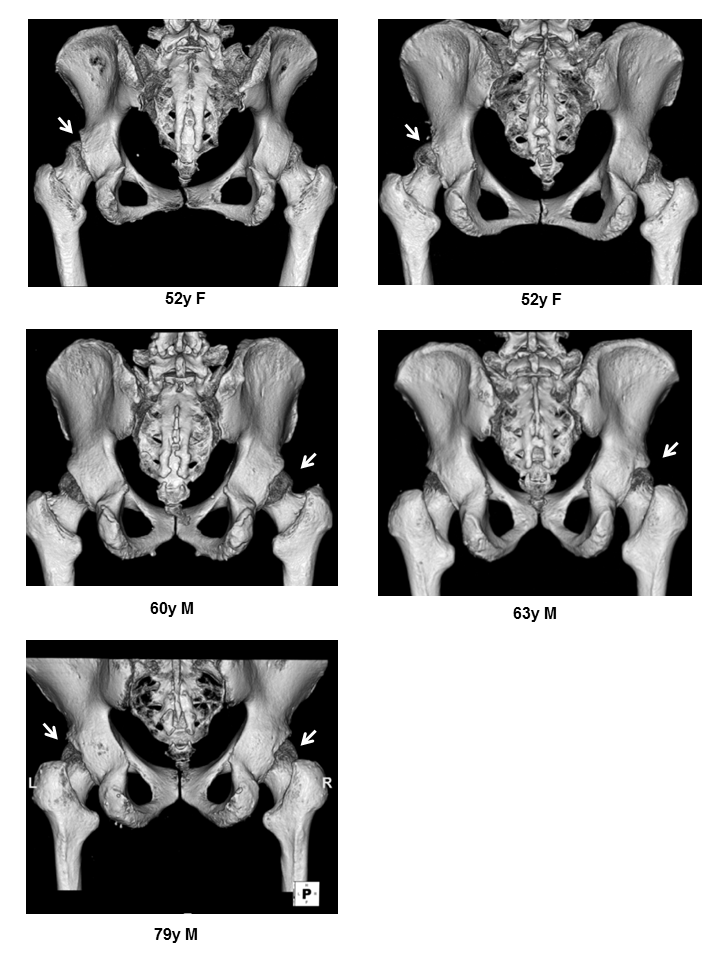

Supplement: Additional file 6: Figure S5. — A posterior view of three-dimensional pelvic CT image in all subjects at 51 years or older with focal concavity of posterior superior acetabulum as indicated by arrows. (TIFF 443 kb) [file 12891_2015_791_MOESM6_ESM.tif]

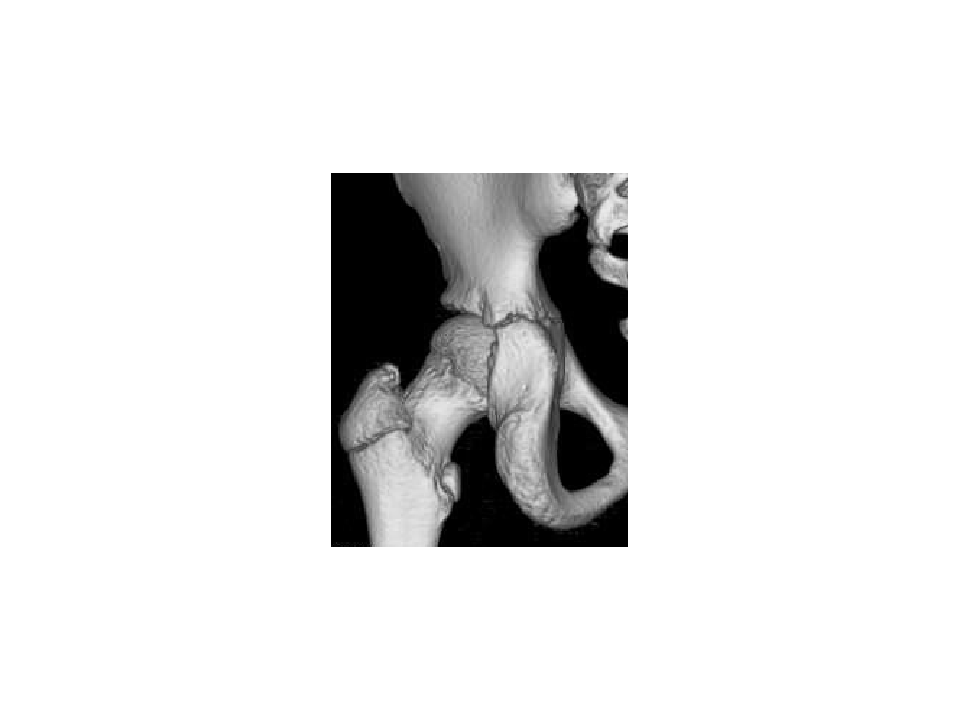

Supplement: Additional file 7: Figure S6. — A posterior view of the representative three-dimensional pelvic CT image during growth, showing the fusion site between ilium and ischium. (TIFF 157 kb) [file 12891_2015_791_MOESM7_ESM.tif]
