# Supplementary material for: Focal concavity of posterior superior acetabulum and its relation with acetabular dysplasia and retroversion in adults without advanced hip osteoarthritis
Source: BMC Musculoskelet Disord. 2015 Nov 2;16:330. doi: 10.1186/s12891-015-0791-z (PMC4631111; doi:10.1186/s12891-015-0791-z)
Supplement: Additional file 2: Table S1. — Inter-rater reliability between two readers. (DOCX 21 kb) [file 12891_2015_791_MOESM2_ESM.docx]

**Additional file 2: Table S1 Inter-rater reliability between two readers**

|  | **All (n=200)** | **50 years or younger (n=98)** |
| --- | --- | --- |
| **Pearson correlation coefficient***  **Lateral center edge angle**  **Tönnis angle**  **Anterior center edge angle**  **Acetabular version angle**  **Intraclass correlation coefficient***  **Lateral center edge angle**  **Tönnis angle**  **Anterior center edge angle**  **Acetabular version angle**  **Cronbach’s alpha***  **Lateral center edge angle**  **Tönnis angle**  **Anterior center edge angle**  **Acetabular version angle** | 0.990  0.984  0.993  0.991  0.990  0.983  0.993  0.991  0.995  0.992  0.996  0.996 | 0.992  0.987  0.995  0.992  0.992  0.986  0.994  0.992  0.996  0.993  0.997  0.996 |

*Analyses using SPSS ver. 22 (IBM Corporatio, Chicago, IL, USA).
